# Preclinical Evaluation of Botulinum Toxin Type E (TrenibotulinumtoxinE) Using the Mouse Digit Abduction Score (DAS) Assay

**DOI:** 10.3390/toxins17050230

**Published:** 2025-05-06

**Authors:** Gregory S. Nicholson, David Canty, Annemarie Southern, Kevin Whelan, Amy D. Brideau-Andersen, Ron S. Broide

**Affiliations:** Allergan Aesthetics, an AbbVie Company, Irvine, CA 92612, USA

**Keywords:** neurotoxin, rodents, paralysis, dose–response relationship

## Abstract

TrenibotulinumtoxinE (trenibotE), a botulinum neurotoxin serotype E (BoNT/E), is being developed for clinical use, and can fill a unique treatment gap for patients who are seeking neurotoxin treatment with a rapid onset and short duration of effect. This preclinical study characterized the pharmacological activity of trenibotE using the mouse Digit Abduction Score (DAS) assay. A comparative analysis was also performed between trenibotE and an equi-efficacious dose of the botulinum neurotoxin serotype A (BoNT/A) onabotulinumtoxinA (onabotA). TrenibotE showed a dose-dependent increase in peak DAS and duration of effect. A comparison of onabotA and trenibotE in this assay at approximate equi-efficacious doses showed trenibotE to have a faster onset of effect (trenibotE yielded a significantly greater effect as early as 6 h post-injection), shorter time to peak effect (24–27 h vs. 2 days), and an overall shorter duration of response (3 days vs. 14 days). The unique temporal characteristics of trenibotE and pharmacological differentiation from onabotA observed in this preclinical assay support the clinical development of this molecule.

## 1. Introduction

Botulinum neurotoxins (BoNTs), which are derived from several strains of Clostridium botulinum, inhibit neurotransmitter release in the neuromuscular junction (NMJ), thus resulting in transitory muscle relaxation [1]. There are 7 different recognized serotypes of BoNT (BoNT/A, B, C1, D, E, F, G) [2]. The most common BoNT serotype that is commercially available is botulinum neurotoxin serotype A (BoNT/A), which is the active ingredient in drug products such as abobotulinumtoxinA, daxibotulinumtoxinA-lanm, incobotulinumtoxinA, onabotulinumtoxinA (onabotA), and prabotulinumtoxinA-xvfs [3]. The pharmacology, efficacy, and safety of onabotA is well characterized in both preclinical [4,5] and clinical settings and has been approved for multiple medical and aesthetic indications in over 100 countries [2,6,7,8]. Unique manufacturing processes, proprietary formulations, and potency assessments for each BoNT product contribute to the non-interchangeability of units [9]. Furthermore, assigned unit values are impacted by inherent pharmacological differences between toxin serotypes [9,10].

More recently, a botulinum neurotoxin serotype E (BoNT/E), trenibotulinumtoxinE (trenibotE; Allergan Aesthetics, an AbbVie Company, Irvine, CA, USA), has been undergoing development for clinical use. Similar to other BoNTs, BoNT/E inhibits neuromuscular transmission through 4 distinct biological processes [1,2,11]. BoNT/E binds to protein receptors on the presynaptic nerve terminal, then enters a synaptic vesicle via receptor-mediated endocytosis. The light chain catalytic domain of BoNT/E translocates across the synaptic vesicle membrane and cleaves synaptosomal-associated protein of 25 kDa (SNAP25), which is part of the soluble N-ethylmaleimide-sensitive factor attachment protein receptors (SNAREs), thereby inhibiting neurotransmitter release. Although the light chains of BoNT/E and BoNT/A both cleave SNAP25, they cleave at different locations on the molecule (BoNT/E cleaves at position 180, while BoNT/A cleaves at position 197) [10,12]. Preclinical and clinical studies have demonstrated that BoNT/E has a unique pharmacological profile, exhibiting a fast onset of effect and short duration compared with BoNT/A [10,12,13,14,15].

This preclinical study aims to characterize the pharmacological activity of trenibotE using the mouse Digit Abduction Score (DAS) assay. The DAS assay is a well-characterized, in vivo preclinical assessment that measures the local muscle-weakening effect of toxins on startle-induced digit abduction (toe spread) following intramuscular toxin injection into the hindlimb skeletal muscle [4]. The DAS assay has been used to evaluate and compare the potency, efficacy, and duration of activity of different BoNT serotypes, including onabotA [4,5,15,16,17]. To complement the current study, a comparative head-to-head analysis between trenibotE and onabotA as well as historical data from approximate equi-efficacious doses (i.e., doses that yield a comparable/similar level of observed effect) are reported. Head-to-head analysis between trenibotE and onabotA was performed to assess onset of effect because historical onabotA data did not assess early timepoints.

## 2. Results

### 2.1. Dose-Dependent Peak Mouse DAS Response and Duration of Effect

**Figure 1:**
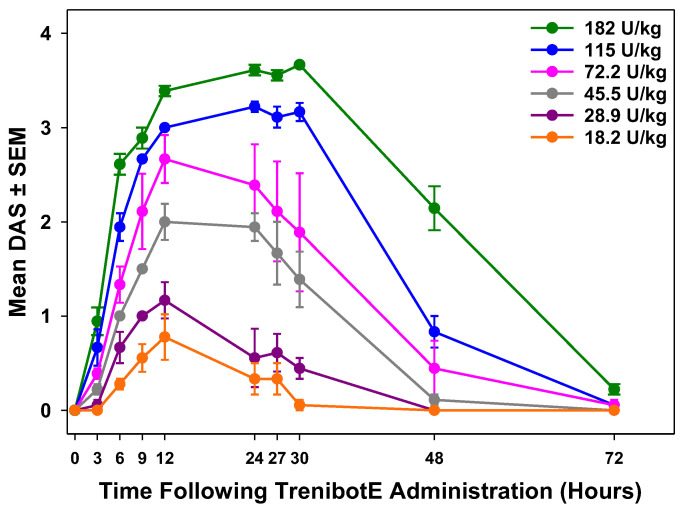
TrenibotE yielded a dose-dependent peak response and duration of effect in the mouse DAS assay. *N* = 3 independent studies; *n* = 6 mice/dose group for each study, for a total of *n* = 18 mice per dose group across the 3 studies. DAS, Digit Abduction Score; SEM, standard error of the mean; TrenibotE, trenibotulinumtoxinE.

TrenibotE showed a dose-dependent increase in the peak DAS and duration of response, with increasing concentrations of toxin leading to decreased digit abduction for longer periods of time (Figure 1). For all trenibotE doses, the peak DAS occurred between 12 and 27 h after injection, with peak responses for lower doses occurring earlier than for the 2 highest doses (Figure 1). In the initial DAS evaluation, the onset of effect (mean DAS > 0) was observed at 6 h post-injection for trenibotE doses of 18.2 and 28.9 U/kg, and as early as 3 h after injection (earliest assessment timepoint) for trenibotE doses of 45.5, 72.2, 115.0, and 182.0 U/kg. The DAS responses for trenibotE doses of 18.2, 28.9, and 45.5 U/kg returned to baseline by 30–48 h after injection, whereas the DAS responses for trenibotE doses of 72.2, 115.0, and 182.0 U/kg returned to baseline by 72 h after injection (final assessment timepoint).

### 2.2. Comparative Analysis Between TrenibotE and OnabotA DAS Response

**Figure 2:**
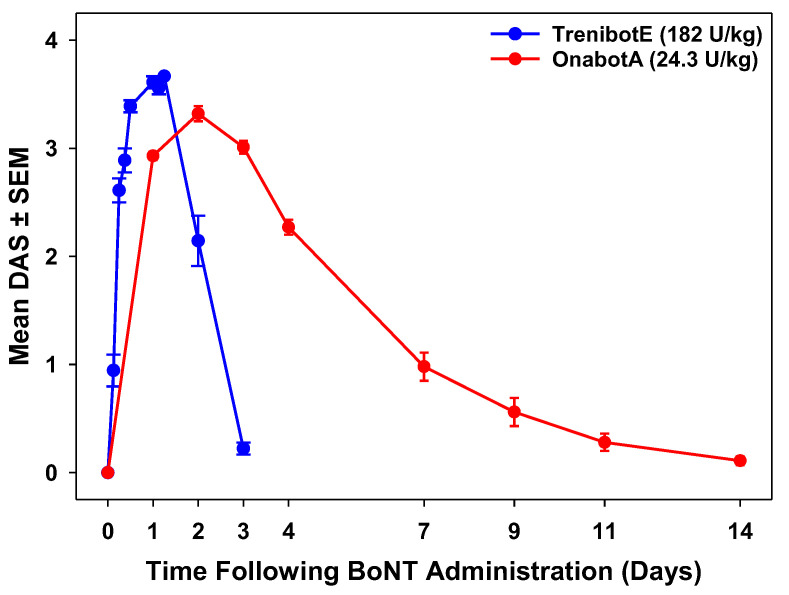
TrenibotE showed a shorter duration of effect compared with onabotA in the mouse DAS assay. The curve shown for trenibotE is the same as that for the 182.0 U/kg dose in Figure 1 but on a different *x*-axis scale. For trenibotE, *N* = 3 independent studies; *n* = 6 mice/dose group for each study, for a total of *n* = 18 mice per dose group across the 3 studies. The onabotA curve reflects previously published historical data [5]. For onabotA, *N* = 9 independent studies; *n* = 6 mice for each study, for a total of *n* = 54 mice across the 9 studies. BoNT, botulinum neurotoxin; DAS, Digit Abduction Score; OnabotA, onabotulinumtoxinA, SEM, standard error of the mean; TrenibotE, trenibotulinumtoxinE.

**Figure 3:**
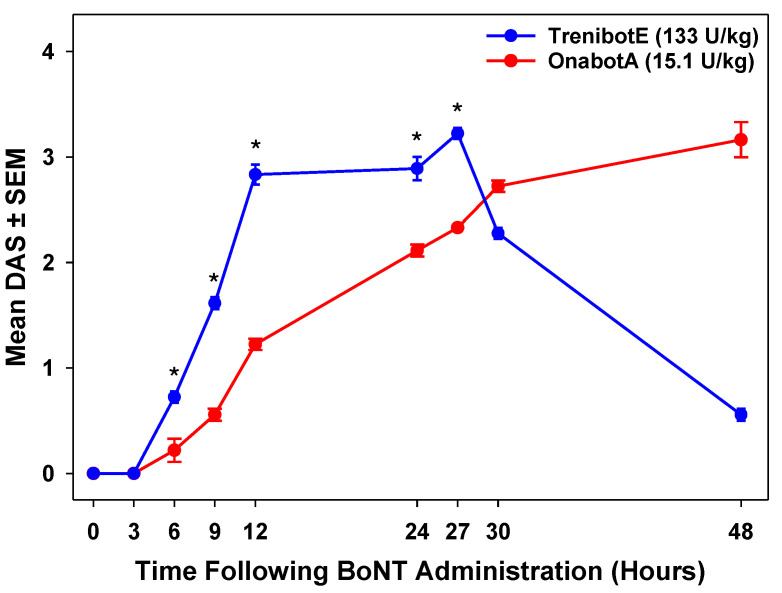
TrenibotE showed a faster onset to peak (27 h) compared with onabotA (48 h) when administered at approximate equi-efficacious dosing (≈ED_75_) in the mouse DAS assay. Here, *N =* 3 independent studies were conducted for trenibotE and onabotA with *n* = 6 mice/dose group for each study, for a total of *n* = 18 mice per dose group across the 3 studies. BoNT, botulinum neurotoxin; DAS, Digit Abduction Score; OnabotA, onabotulinumtoxinA; SEM, standard error of the mean; TrenibotE, trenibotulinumtoxinE. * Indicates a significant difference (*p* < 0.05 using Student’s and Welch’s *t* tests).

In the initial mouse DAS evaluation, the response for the highest dose of trenibotE (182.0 U/kg) was compared with historical mouse DAS data for an approximate equi-efficacious dose of onabotA (24.3 U/kg) [5]. Compared with historical data for onabotA, trenibotE showed an earlier peak response and an overall shorter duration of response (Figure 2). In this comparison, the trenibotE peak effect was observed at 24 h post-injection at an approximate 90% effective dose (ED90) level of effect, and then returned to baseline by 72 h post-injection. In contrast, the peak effect for onabotA occurred 48 h post-injection at an approximate ED85 level of effect, and then returned to baseline by 2 weeks post-injection. To further interrogate and compare the onset of effect of trenibotE and onabotA, an additional DAS assay was performed, focusing on early timepoints at approximate equi-efficacious doses for both toxins (≈ED_75_; 133 U/kg for trenibotE, 15.1 U/kg for onabotA). The onset of effect (mean DAS > 0) for trenibotE and onabotA in the follow-up mouse DAS occurred as early as 6 h post-injection for both (earliest assessment timepoint was 3 h), but the mean DAS was significantly higher for trenibotE (*p* < 0.05) at each timepoint from 6 to 27 h (time of peak effect for trenibotE; Figure 3). The observed peak effect for onabotA in this follow-up DAS evaluation was 48 h, the same as in the evaluation presented in Figure 2. These data demonstrate that trenibotE has a faster onset to peak effect than onabotA at equi-efficacious dosing.

## 3. Discussion

Although BoNTs are widely used in the clinic, there is a need in aesthetic medicine for botulinum neurotoxins that provide a fast onset and a short duration of clinical effect. This type of treatment can address an unmet need in patients seeking rapid treatment results or who are hesitant to initiate BoNT/A treatment due to clinical effects lasting many months. In this study, using the mouse DAS assay, trenibotE showed a dose dependency in both peak response and duration of effect. Furthermore, it showed a faster onset of effect and shorter duration of effect compared with an approximate equi-efficacious dose of onabotA.

This study supports observations from previous exploratory preclinical and clinical studies comparing BoNT/E with BoNT/A [10,12,13,14,15]. In one particular study, it was demonstrated that BoNT/A was more potent than BoNT/E in both mouse and rat DAS assays [14]. In situ contraction measurements in rat extensor digitorum longus (EDL) muscles demonstrated faster recovery of muscle tension after BoNT/E (20 mouse units [MU]) exposure versus BoNT/A (5 LD_50_ MU) [13]. On day 7 post-injection, muscles injected with BoNT/E, but not BoNT/A, showed recovery of muscle tension (67% vs. 2% recovery, respectively) [13]. By day 15, BoNT/E showed complete recovery of muscle tension compared with BoNT/A (100% vs. 11% recovery, respectively) [13]. The BoNT/E and BoNT/A doses used in this study were selected because they produced near total paralysis and total paralysis of the injected muscles within 24 and 48 h, respectively, without systemic toxicity [13]. Variations in the BoNT doses and overall methodology between this previous study [13], which evaluated in situ muscle contraction, and the present study, which evaluated in vivo muscle function (digit abduction), likely account for the observed differences in the duration of response between BoNT/E and BoNT/A. Differences in duration were also demonstrated in a rat version of the DAS model; however, a limitation of that study was that the authors drew conclusions about duration differences between BoNT/E and BoNT/A, but did not administer the 2 toxins at equi-efficacious doses [15].

In a small clinical study, 11 participants were injected with either BoNT/E or BoNT/A (3 international units per participant) in each extensor digitorum brevis (EDB) muscle to assess recovery of NMJ function from BoNT-induced paralysis, which was measured using the mean percentage compound muscular action potential amplitude (%CMAP) [10]. EDB muscles treated with BoNT/E recovered more rapidly than the contralateral muscle treated with BoNT/A [10]. By 30 days post-injection, BoNT/E-treated muscles showed significant recovery versus BoNT/A-treated muscles (%CMAP value of >60% vs. >20%, respectively; *p* < 0.001), and by 90 days, BoNT/E-treated but not BoNT/A-treated muscles recovered back to baseline (%CMAP value of 100% vs. >40%, respectively; *p* < 0.001) [10]. In an exploratory, dose-escalation clinical study (*N* = 42) evaluating BoNT/E for the treatment of moderate to severe glabellar frown lines, most dose groups achieved the primary endpoint (2-grade improvement on the investigator-rated Facial Wrinkle Scale [FWS] at any post-baseline visit through day 30) [18], with the 2 highest-dose groups showing responder rates of 80%, compared with 40% to 60% for the intermediate-dose groups [18]. The onset of clinical effect was observed as early as 24 h post-injection (first assessment timepoint), and the duration of effect (defined as the proportion of responders with a rating of “none” or “mild” on the FWS) was observed between 14 and 30 days for the highest doses [18]. Because of the limited understanding regarding how BoNT units were assigned in these studies, the BoNT doses should not be extrapolated to the current preclinical study.

Variations in the structure and pharmacology between BoNT/E and BoNT/A may contribute to differences in the onset and duration of effect. Unlike BoNT/A, where the 3 functional domains (binding, translocation, and catalytic domains) have a “linear” or “open” configuration, BoNT/E has been shown to have a “closed” configuration. This arrangement may be associated with a more rapid translocation rate for trenibotE versus onabotA across the endosomal compartment, resulting in a faster onset of effect [3,19,20]. Furthermore, although BoNT/E and BoNT/A both specifically bind to the glycosylated synaptic vesicle protein 2 (SV2) in the presynaptic nerve terminal, BoNT/E is selective for only 2 of 3 homologous isoforms of SV2 (SV2A and SV2B), whereas BoNT/A recognizes all 3 isoforms (SV2A, SV2B, and SV2C), preferentially binding to SV2C [1,20]. Thus, variations in tissue distribution patterns of the SV2 isoforms may also contribute to the different temporal and biological characteristics of BoNT/E compared with BoNT/A, as well as assigned units of activity.

The shorter duration of effect of BoNT/E compared with BoNT/A may result from differences in the cleavage pattern of SNAP25 and/or shorter intraneuronal half-life of the light chain of the BoNT/E molecule, which cleaves SNAP25 [1,21,22]. BoNT/E and BoNT/A have been reported to cleave different sites of SNAP25, thus removing 26 or 9 amino acid residues, respectively, from the C-terminus [23]. The cleavage of additional residues from SNAP25 by BoNT/E may result in a more rapid replenishment of functional SNAP25, leading to a shorter duration of effect compared with BoNT/A-cleaved SNAP25, which has a prolonged half-life within the synaptic terminal [24,25]. The light chain of BoNT/E may also be more susceptible to degradation than BoNT/A because of differences in subcellular localization. The light chain of BoNT/E has been shown to be localized to the cytosol versus the light chain of BoNT/A, which has been shown to reside in the plasma membrane, where it may associate with a slow recycling compartment [22]. Unlike BoNT/E, the BoNT/A light chain has also been demonstrated to associate with septin cytoskeletal proteins, which protect it from intracellular degradation [26], and the de-ubiquitinating enzyme VCIP135, which protects it from proteasomal degradation [27].

A limitation of this study is that the historical onabotA data used for comparison did not yield the same peak effect as trenibotE. Although the peak responses were close (≈ED85 for onabotA vs. ≈ED90 for trenibotE), they were not of the same level. However, even with the lower observed peak response for onabotA vs. trenibotE, the BoNT/A serotype still yielded an obvious and substantial extension in duration. This further exemplifies the differing pharmacology for these 2 distinct proteins in the current in vivo model. In the follow-up mouse DAS study, we observed that trenibotE (133 U/kg) and onabotA (15.1 U/kg) administered at equi-efficacious doses had an onset of effect as early as 6 h. However, the mean DAS values for trenibotE were significantly higher (*p* < 0.05) than those observed for onabotA at all timepoints from 6 to 27 h (observed peak for trenibotE).

Aside from inherent molecular and pharmacological differences between BoNT/E and BoNT/A, there may be differences in their muscle diffusion profiles, which can influence toxin efficacy [15,28]. Furthermore, differences in NMJ arborization patterns between different muscles can also be a key anatomical factor affecting toxin clinical potency and duration of action [29,30]. As such, knowledge of specific muscle neuroanatomy and injection technique can be useful for obtaining optimal toxin clinical efficacy [31,32].

Different unit dosing is required for onabotA and trenibotE, which reflects inherent differences in pharmacology, including different LD_50_ values, between the 2 distinct serotypes. Likewise, due to proprietary formulations and potency assessments, units of BoNT products are not interchangeable. Therefore, comparing the units of trenibotE with other BoNT/E materials evaluated in preclinical and/or clinical studies, such as those previously described above, is not feasible [9]. Future preclinical and clinical studies will provide more direct and comprehensive comparisons between trenibotE and onabotA.

## 4. Conclusions

TrenibotE yielded dose-dependent peak DAS and duration of effect profiles in the mouse DAS assay. Relative to head-to-head and historical onabotA data, trenibotE showed a faster onset and overall shorter duration of effect when administered at approximate equi-efficacious doses. TrenibotE fills a unique treatment gap for patients who are seeking neurotoxin treatment with a rapid onset and short duration of effect. The unique temporal characteristics of trenibotE and differentiation in pharmacology from onabotA support the clinical development of this molecule.

## 5. Materials and Methods

### 5.1. TrenibotE and OnabotA Preparation

Working solutions for the trenibotE drug substance (DS) full-dose response evaluation were prepared via serial dilution with human serum albumin/0.9% saline to yield final concentrations of 18.2, 28.9, 45.4, 72.2, 115.0, and 182.0 U/kg. For the onset evaluation versus onabotA, trenibotE DS was prepared in the same manner to yield a final concentration of 133 U/kg, and onabotA drug product was reconstituted in 0.9% saline to yield a final concentration of 15.1 U/kg. Two different lots of BoNT/E were utilized for this study. For all toxins, potency was determined either by mouse LD_50_ bioassay or by cell-based potency assay.

### 5.2. Mice

Female CD-1 mice (Charles River Laboratories International, Inc., Raleigh, NC, USA) weighing between 21 and 28 g (range for a given experimental replicate was within 4 g) were group-housed and maintained on a 12-h light–dark cycle, with food and water provided ad libitum. All procedures were approved by the Allergan/AbbVie Institutional Animal Care and Use Committee (IACUC), following guidelines put forward by the American Association for Laboratory Animal Science (AALAS).

### 5.3. Mouse DAS Assay

The mouse DAS assay has been previously described [4,17]. On day 0 of the experiment, mice were weighed, prescreened for a DAS = 0 (i.e., normal response), and randomized to trenibotE and onabotA dose groups. Mice were given a 5 μL intramuscular injection of trenibotE or onabotA into the right gastrocnemius muscle using a 30-gauge 0.5-inch needle attached to a 250-μL glass syringe. Contralateral legs were not injected and served as an untreated visual reference when scoring mice. Solutions were masked by a separate investigator. To assess the DAS response, mice were suspended by the tail to elicit a classic startle response wherein the animal extends its hind limbs and abducts (spreads) its hind digits. Toxin-induced muscle paralysis was scored using the 5-point ordinal DAS scale (scored 0–4), where a score of “0” represented a normal response, and a score of “4” represented maximum reduction in digit abduction.

All dose groups were rated on the DAS scale and observed for any changes in general activity level within the home cage at 3, 6, 9, 12, 24, 27, 30, 48, and 72 h post-injection, in accordance with the IACUC-approved criteria for assessing the clinical well-being of a test animal. If all mice in a group were scored as DAS = 0 for 2 consecutive observation days, indicating a return to baseline, DAS ratings and observations of general activity ceased. Peak response and duration of response curves were generated from the mean DAS across 3–6 independent studies (*n* = 6 mice/dose group for each study, for a total of *n* = 18–36 mice per dose group). Investigators and observers were blinded to the doses.

### 5.4. OnabotA Historical Data

The DAS assay performed on mice injected with onabotA has been previously described [5]. Briefly, female CD-1 mice were given a 5-μL intramuscular injection of onabotA into the right gastrocnemius muscle using a 30-gauge needle. The mouse DAS assay was performed on days 0, 1, 2, 3, 4, 7, 9, 11, and 14 post-injection. Three separate lots of onabotA were tested in triplicate (*N* = 9 independent studies; *n* = 6 mice for each study, for a total of *n* = 54 mice). For the current analysis, a 24.3-U/kg dose of onabotA, representing a dose causing an approximate 85% reduction in digit abduction, was selected as an equi-efficacious dose for comparison with 182.0 U/kg of trenibotE.

### 5.5. Statistical Analyses

For the comparison of onset of effect (first observation of DAS > 0), mean DAS values for trenibotE and onabotA at the 3-, 6-, 9-, 12-, 24-, and 27-h timepoints were compared using Student’s and Welch’s *t* tests to determine whether there were any differences, with statistical significance defined as *p* < 0.05.

## Data Availability

AbbVie is committed to responsible data sharing regarding the clinical trials we sponsor. This includes access to anonymized, individual and trial-level data (analysis data sets), as well as other information (e.g., protocols and Clinical Study Reports, or analysis plans), as long as the trials are not part of an ongoing or planned regulatory submission. This includes requests for clinical trial data for unlicensed products and indications. This clinical trial data can be requested by any qualified researchers who engage in rigorous, independent scientific research, and will be provided following review and approval of a research proposal and Statistical Analysis Plan (SAP) and execution of a Data Sharing Agreement (DSA). Data requests can be submitted at any time after approval in the US and Europe and after acceptance of this manuscript for publication. The data will be accessible for 12 months, with possible extensions considered. For more information on the process, or to submit a request, visit the following link: https://vivli.org/ourmember/abbvie/then select “Home”.
